# Identification of a Ferroptosis-Related Signature Model Including mRNAs and lncRNAs for Predicting Prognosis and Immune Activity in Hepatocellular Carcinoma

**DOI:** 10.3389/fonc.2021.738477

**Published:** 2021-09-09

**Authors:** Zi-An Chen, Hui Tian, Dong-Mei Yao, Yuan Zhang, Zhi-Jie Feng, Chuan-Jie Yang

**Affiliations:** Department of Gastroenterology, The Second Hospital of Hebei Medical University, Shijiazhuang, China

**Keywords:** hepatocellular carcinoma, LASSO regression analysis, ferroptosis-related mRNA and lncRNAs, tumor microenvironment, immune infiltration

## Abstract

**Background:**

Ferroptosis is a novel form of regulated cell death involved in tumor progression. The role of ferroptosis-related lncRNAs in hepatocellular carcinoma (HCC) remains unclear.

**Methods:**

RNA-seq and clinical data for HCC patients were downloaded from The Cancer Genome Atlas (TCGA) Genomic Data Commons (GDC) portal. Bioinformatics methods, including weighted gene coexpression network analysis (WGCNA), Cox regression, and least absolute shrinkage and selection operator (LASSO) analysis, were used to identify signature markers for diagnosis/prognosis. The tumor microenvironment, immune infiltration and functional enrichment were compared between the low-risk and high-risk groups. Subsequently, small molecule drugs targeting ferroptosis-related signature components were predicted *via* the L1000FWD and PubChem databases.

**Results:**

The prognostic model consisted of 2 ferroptosis-related mRNAs (SLC1A5 and SLC7A11) and 8 ferroptosis-related lncRNAs (AC245297.3, MYLK-AS1, NRAV, SREBF2-AS1, AL031985.3, ZFPM2-AS1, AC015908.3, MSC-AS1). The areas under the curves (AUCs) were 0.830 and 0.806 in the training and test groups, respectively. Decision curve analysis (DCA) revealed that the ferroptosis-related signature performed better than all pathological characteristics. Multivariate Cox regression analysis showed that the risk score was an independent prognostic factor. The survival probability of low- and high-risk patients could be clearly distinguished by the principal component analysis (PCA) plot. The risk score divided HCC patients into two distinct groups in terms of immune status, especially checkpoint gene expression, which was further supported by the Gene Ontology (GO) biological process, and Kyoto Encyclopedia of Genes and Genomes (KEGG) analysis. Finally, several small molecule drugs (SIB-1893, geldanamycin and PD-184352, etc) targeting ferroptosis-related signature components were identified for future reference.

**Conclusion:**

We constructed a new ferroptosis-related mRNA/lncRNA signature for HCC patients. The model can be used for prognostic prediction and immune evaluation, providing a reference for immunotherapies and targeted therapies.

## Introduction

According to epidemiological studies, hepatocellular carcinoma (HCC) is considered the seventh most common malignancy and the second most common cause of cancer-related death (1). Many factors, including chronic infection with HBV/HCV, alcohol abuse, long-term obesity or exposure to aflatoxin, have been reported to be related to the progression of HCC (2). Considering that the early monitoring methods for HCC are still limited and that the tumor easily metastasizes and has a poor prognosis, it is necessary to develop new detection methods and identify new therapeutic targets for HCC.

Ferroptosis is a unique form of regulated cell death associated with iron metabolism, which is different from apoptosis, necrosis, and autophagy (3). Although the detailed mechanism underlying the role of ferroptosis in tumors is still unclear, several studies have reported that ferroptosis is involved in various cancers, including breast cancer (4–6), pancreatic cancer (7), ovarian cancer (8), and HCC (9–12). Compared to normal nontumor cells, cancer cells have a higher level of iron, which indicates the potential of ferroptosis inducers in new antitumor strategies (13, 14). For example, the triterpene saponin ardisiacrispin B and epunctanone exert cytotoxic effects on cancer cells with multiple drug resistance partly *via* ferroptosis (15, 16). In addition to ferroptosis-inducing agents, an increasing number of ferroptosis-related genes have been identified and found to be involved in the progression of cancers by serving as mediators of ferroptosis-related pathways. In HCC, CISD1 and a polymorphism of the TP53 gene (the S47 variant) are reported to negatively regulate ferroptosis, which proves that ferroptosis-related genes play a role in tumor progression (17, 18). In addition, several ferroptosis-related genes, including MI1G, NRF2, and Rb, were found to protect HCC cells from sorafenib-induced ferroptosis (19–21).

Based on the existing findings, we have noticed that ferroptosis plays a pivotal role in the progression of HCC; however, the specific function of ferroptosis-related long noncoding RNAs (lncRNAs) in HCC has not been fully elucidated. lncRNAs are a class of noncoding transcripts more than 200 nucleotides in length (22). It has been proved that lncRNAs serve as pivotal players in posttranscriptional regulatory mechanisms that target mRNA splicing, stability, or translation, the scope of which is still expanding (23). Dynamic alterations in the expression and mutation of lncRNAs are closely associated with tumorigenesis, tumor progression, metastasis, and cancer immunity indicating the emerging roles of lncRNAs as new biomarkers and therapeutic targets for cancer treatment strategies (24–26). Therefore, investigating lncRNAs related to ferroptosis and HCC is essential to our understanding of the mechanisms of tumor development. Recently, a model containing 3 ferroptosis-related lncRNAs was reported; however, it exhibited low predictive power for HCC with an area under the curve (AUC)=0.7 (27).

In this study, we constructed a new ferroptosis-related signature including mRNAs and lncRNAs by both weighted gene co-expression analysis (WGCNA) and least absolute shrinkage and selection operator (LASSO) regression analysis. We evaluated the predictive value of the ferroptosis-related signature and investigated the differential immune response in a variety of ways, including tumor microenvironment (TME) analysis and single-sample gene set enrichment analysis (ssGSEA). Furthermore, functional enrichment analysis was performed to clarify the biological functions of these differentially expressed genes (DEGs). Based on the DEG results, several small molecule drugs targeting ferroptosis-related signature components were identified *via* the L1000FWD database, and SIB-1893, geldanamycin and PD-184352 were visualized by PubChem.

## Material and Methods

### Data Collection and Preprocessing

**Figure 1:**
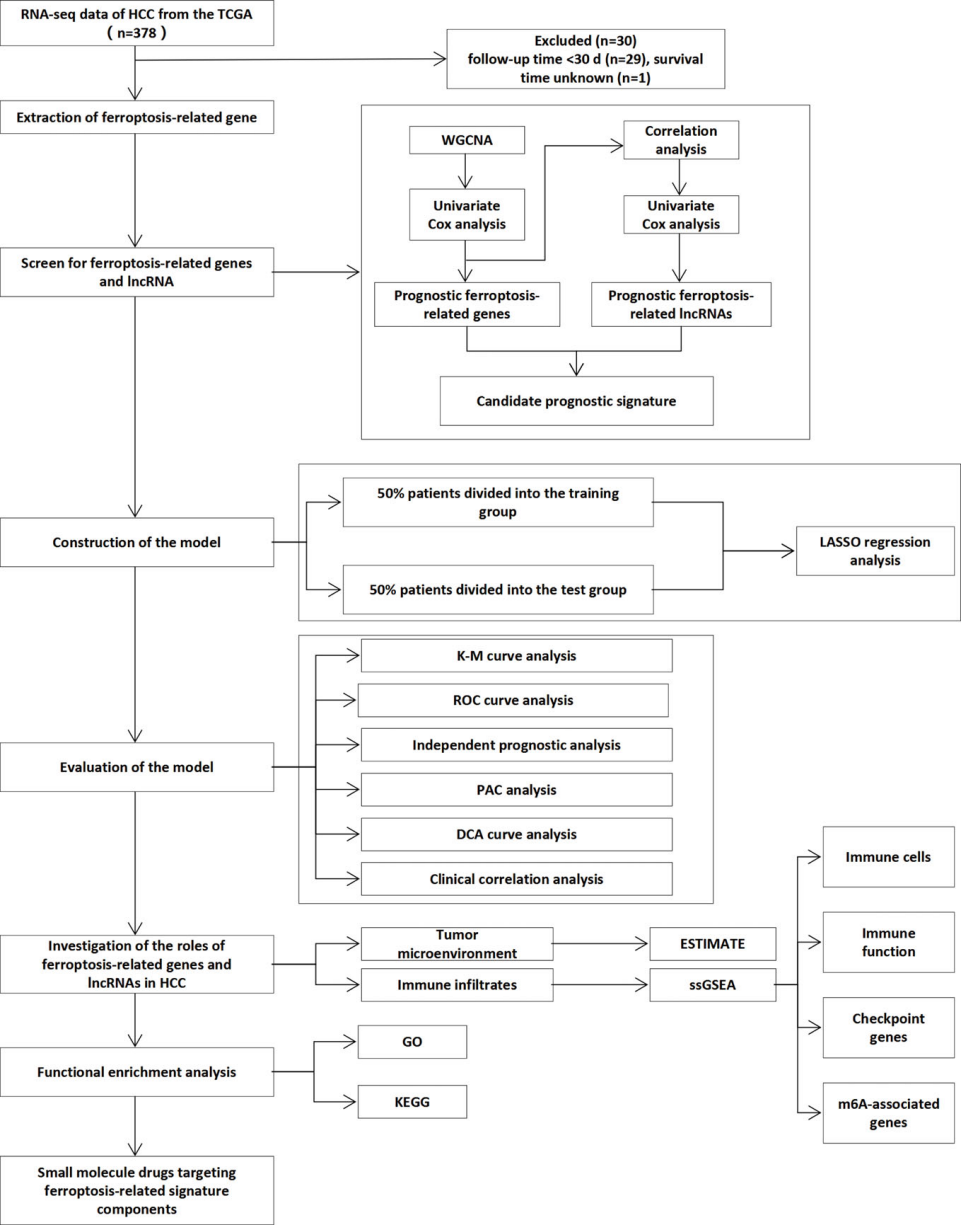
Flowchart of overall study design.

The RNA sequencing (FPKM) and clinical data of HCC patients were downloaded from The Cancer Genome Atlas (TCGA) Genomic Data Commons (GDC) portal (https://portal.gdc.cancer.gov/repository). To reduce errors caused by confounding factors, we exclude samples with patient follow-up time < 30 d (n = 29) and without survival information (n = 1). Detailed information on the clinical data of the 377 samples is shown in **Table 1**. A list of 267 ferroptosis-related genes was compiled based on the FerrDb website (http://www.zhounan.org/ferrdb/) and previous literature (12, 13, 28–30) (**Supplementary Table 1**). The flowchart of this research is exhibited in **Figure 1**.

**Table 1:** Clinical characteristics of patients in the TCGA LIHC cohort.

Characteristic		N = 348
Age	Median	61
	Range	16-90
	Male	238
Sex	Female	110
	G1	53
	G2	164
Grade	G3	113
	G4	13
	NA	5
	Stage I	165
	Stage II	78
Clinical stage	Stage III	81
	Stage IV	3
	NA	21
	T1	172
	T2	85
T stage	T3	75
	T4	13
	NA	3
	M0	250
M stage	Ml	3
	NA	95
	NO	T6
N stage	N1	3
	NA	101
	Alive	223
Vital status	Dead	125

### WGCNA

WGCNA was performed to identify coexpressed gene modules and investigate the relationships between gene networks and clinical traits. WGCNA was conducted with the “WGCNA” package. Pearson correlation tests were performed to construct a matrix to establish the module-trait relationships between ferroptosis-related genes and vital status according to the β value (soft-threshold value). The modules with a *p*<0.05 were considered associated with vital status and were selected for further research.

### Construction of the LASSO Cox Regression Model and Survival Analysis

Before establishing the model, the ferroptosis-related genes from WGCNA were tested by univariate Cox regression analysis (*p <*0.001). Furthermore, ferroptosis-related lncRNAs co-expressed with ferroptosis-related genes were screened by Pearson correlation test (correlation coefficient >0.4, and *p <*0.001). The lncRNAs were further screened by univariate Cox regression analysis (*p <*0.001). Then, the selected ferroptosis-related genes and lncRNAs were merged to establish the model. A network containing the ferroptosis-related mRNA-lncRNA network was constructed and visualized by Cytoscape (version 3.7.2)

HCC patients from the TCGA liver hepatocellular carcinoma (LIHC) cohort were randomly divided into a training group, and another 50% were set as the test group. The LASSO Cox regression algorithm was applied to select the ferroptosis-related signature. Finally, a formula for the risk score was established, and we calculated the risk score for each patient as follows:

$$\text{RiskScore}=\sum_{i=1}^{\text{n}}\text{Coe}{\text{f}}_{\text{i}}\times {\text{X}}_{\text{i}}$$

Coefi indicates the correlation coefficient of each ferroptosis-related signature, and X indicates the level of gene expression. The median risk score in the training cohort was set as the cutoff value, and the training group and test group were divided into high-risk and low-risk groups according to the cutoff.

### Determination of Immune Score, Stromal Score, and ESTIMATE Score

The Estimation of STromal and Immune cells in MAlignant Tumors using Expression data (ESTIMATE) algorithm was used to evaluate the ratio of the immune-stromal component in the TME by utilizing the “estimate” R package, which calculated three scores: the immune score (representing the level of immune cell infiltration), stromal score (representing the amount of stroma), and ESTIMATE score (representing the sum of both). A higher score indicated a larger ratio of the corresponding component in the TME.

### Estimation of the Immune Cell Infiltration

To evaluate immune cell infiltration, ssGSEA was used to quantify the tumor-infiltrating immune cell subgroups and immune function between the two groups. The expression of potential immune checkpoint and m6A genes was also determined according to previous literature.

### Functional and Pathway Enrichment Analysis

The DEGs between the low- and high-risk groups were then screened out by the “limma” package using the criteria false discovery rate (FDR) < 0.05 and |log2 fold change (FC)| ≥ 1. We then applied the “limma” and “clusterProfiler” packages to perform Gene Ontology (GO) analysis and Kyoto Encyclopedia of Genes and Genomes (KEGG) pathway enrichment analysis.

### Identification of Potential Compounds

DEGs based on the ferroptosis-related signature were divided into up- and downregulated gene groups. The two groups of genes were then uploaded to the L1000FWD website (https://maayanlab.cloud/L1000FWD/), and then permuted results were obtained. The results were further visualized by the PubChem website (pubchem.ncbi.nlm.nih.gov).

### Statistical Analysis

The data were analyzed with R software version 4.0.4. For comparisons, data conforming to normal and nonnormal distributions were assessed using the unpaired Student’s t-test and the Wilcoxon test, respectively, and the statistical significance threshold was set at *p <*0.05. The survival of HCC patients based on the ferroptosis-related signature was assessed using Kaplan-Meier survival analysis. The receiver operating characteristic curve (ROC) and decision curve analysis (DCA) were performed with the timeROC and ggDCA packages, respectively.

## Results

### Preprocessing of RNA Sequencing Data and Clinical Data

The RNA sequencing and clinical data of HCC patients were downloaded from the TCGA GDC portal on April 30, 2021 (https://portal.gdc.cancer.gov/repository). After data curation, 30 cases were removed from the dataset (29 cases for survival time <30 d, 1 case for lack of survival information). The clinical data for 348 cases are listed in **Table 1**. A total of 267 ferroptosis-related genes were extracted from HCC patient datasets for further WGCNA.

### Identification of Modules Associated With Survival Traits by WGCNA

**Figure 2:**
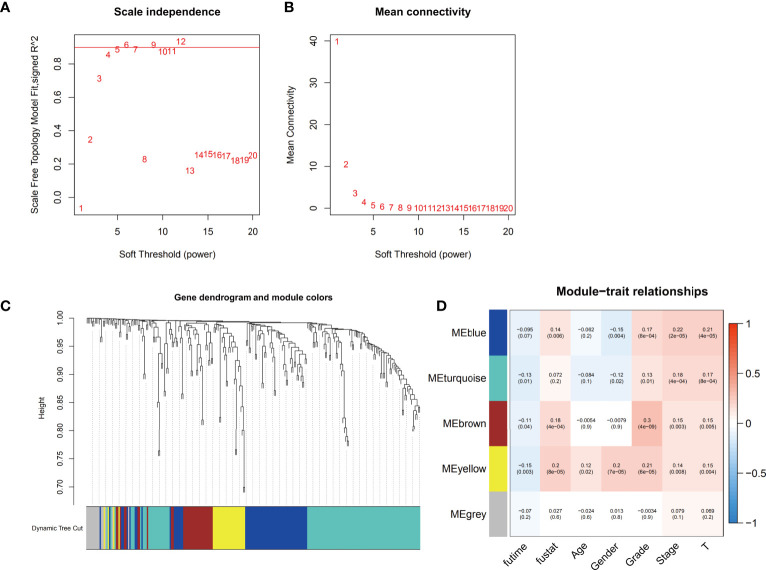
Identification of the weighted gene coexpression network analysis (WGCNA) hub module and assessment of module-trait relationships. **(A, B)** Soft threshold selection to identify the WGCNA module. **(C)** The cluster dendrogram of 5 colored modules based on a dissimilarity measure (1-TOM) in HCC patients. **(D)** Correlation between the gene module and clinical traits, including survival time (futime), vital status (fustat), age, gender, grade, stage and T stage.

The gene coexpression networks of the TCGA-LIHC dataset were established *via* the WGCNA package and are shown in **Figure 2**. To establish scale-free networks, the soft thresholding power was set to β=5 based on scale independence and mean connectivity (**Figures 2A, B**). The dynamic tree cut package was used to generate a gene cluster dendrogram containing 5 co-expression models (**Figure 2C**). The coexpression models are shown in blue, turquoise, brown, yellow and gray and contain 63, 110, 32, 28, and 15 genes, respectively (**Supplementary Table 2**). *p <*0.05 was considered to indicate a significant module-trait relationship between ferroptosis-related genes and vital status (**Figure 2D**). Based on these analyses, the blue, brown, yellow modules containing 123 genes were selected for further analysis.

### Identification of Prognostic Ferroptosis-Related Genes and lncRNAs

According to the univariate Cox regression analysis, a total of 38 prognostic ferroptosis-related genes were screened. Then, by ferroptosis-related lncRNA co-expression analysis, we identified 526 ferroptosis-related lncRNAs (*p <*0.001, correlation coefficient >0.4). Univariate Cox regression analysis was further performed, and 70 prognostic ferroptosis-related lncRNAs were screened.

### Construction and Validation of the LASSO-Cox Model

**Figure 3:**
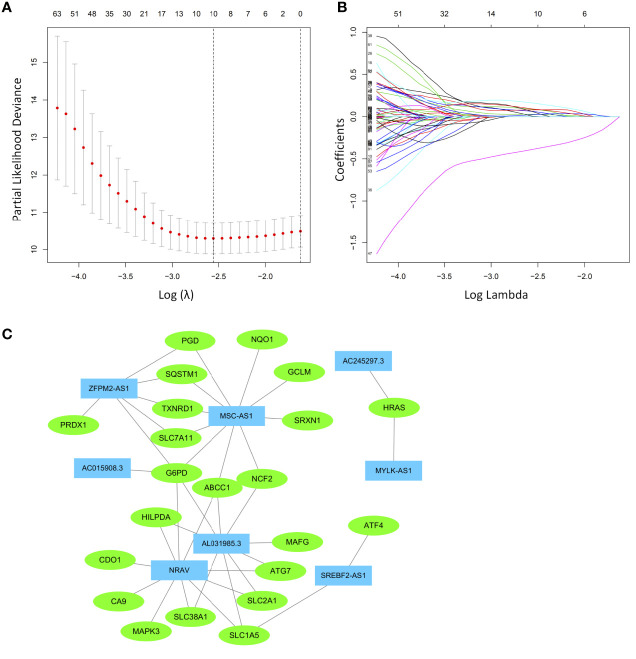
Least absolute shrinkage and selection operator (LASSO) Cox regression analysis was conducted based on the ferroptosis-related signature. **(A)** LASSO coefficient profiles. **(B)** Candidate ferroptosis-related genes and lncRNAs from the univariate Cox regression analysis were filtered by the LASSO algorithm. **(C)** The network containing ferroptosis-related lncRNAs and mRNAs was visualized with Cytoscape. The green nodes indicate ferroptosis-related mRNAs, while the blue nodes indicate ferroptosis-related lncRNAs.

The prognostic ferroptosis-related genes and lncRNAs were merged and served as candidates for establishing the LASSO model (**Supplementary Table 3**). The HCC cohort was randomly divided into an equal training group and a test group. After the model reached the minimum lambda, a prognostic ferroptosis-related signature with 10 components was built (**Figures 3A, B**). In the training group, the median risk score classified patients into a high-risk group and a low-risk group and was calculated as follows: risk score = [expression level of AC245297.3×(0.003128)] + [expression level of MYLK-AS1×(0.086099)] + [expression level of NRAV×(0.061838)] + [expression level of SREBF2-AS1×(0.059227)] + [expression level of AL031985.3×(0.079650)]] + [expression level of ZFPM2-AS1×(0.028512)] + [expression level of AC015908.3×(-0.38804)] + [expression level of MSC-AS1×(0.161449)] + [expression level of SLC1A5×(0.023153)] + [expression level of SLC7A11×(0.090097)]. The correlations between ferroptosis-related genes and lncRNAs were used to construct a network, which was visualized in Cytoscape (**Figure 3C**). Among these, MSC-AS1, ZFPM2-AS1, NRAV and AL031985.3 have a co-expression relationship with more ferroptosis-related genes.

### Prognostic Value of the Ferroptosis-Related Signature Model in the Training and Test Groups

**Figure 4:**
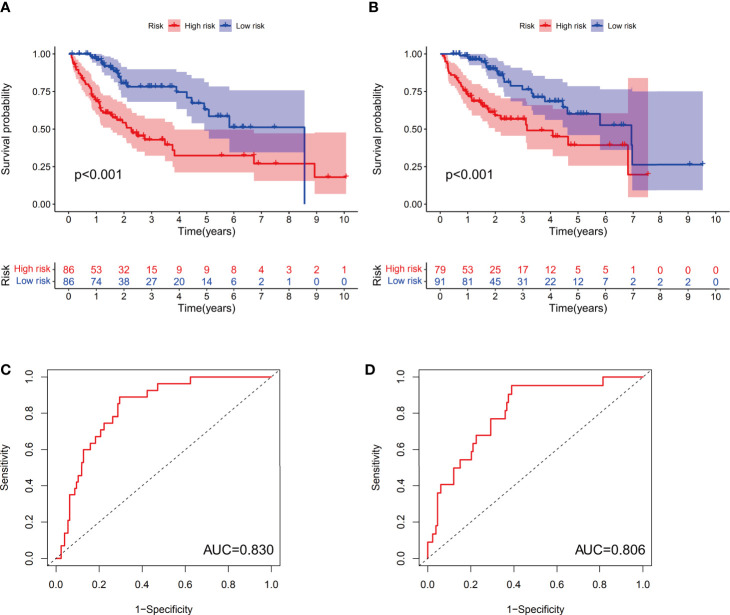
The predictive value of the prognostic model in both the training and test groups. Kaplan-Meier curves were depicted based on the ferroptosis-related signature scores in the training group **(A)** and test group **(B)**. The receiver operating characteristic (ROC) curves of 1-year survival for HCC patients in the training group **(C)** and test group **(D)**.

Examination of the survival curves for the low-risk and high-risk patient groups was performed using the Kaplan-Meier method in both the training and test cohorts. The results from both cohorts showed that patients in the high-risk group had a statistically lower probability of survival (p<0.001 in both cohorts) (**Figures 4A, B**). The AUC for 1-year overall survival (OS) was 0.830 in the training cohort (**Figure 4C**) and 0.806 in the test cohort (**Figure 4D**).

### The Ferroptosis-Related Signature is an Independent Prognostic Factor for HCC

**Figure 5:**
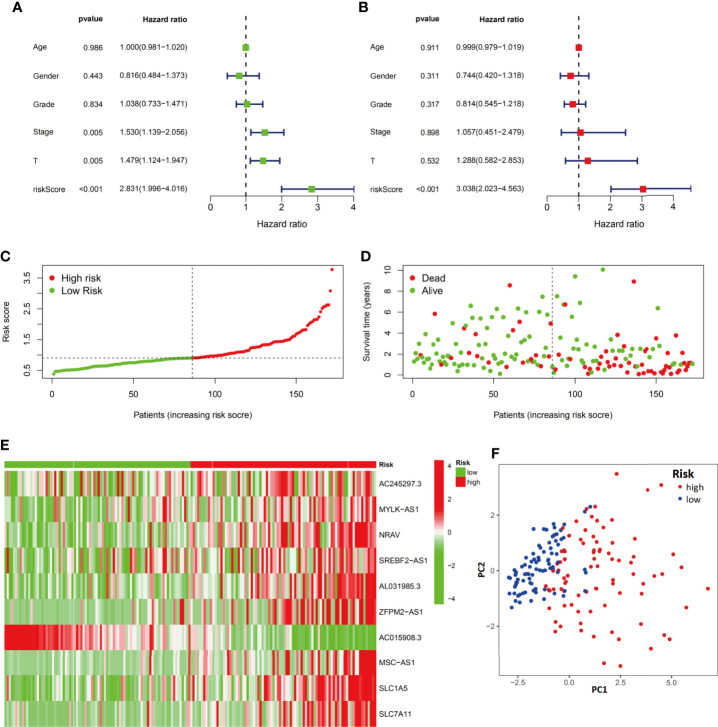
Evaluation of the ferroptosis-related signature in the training group. Independent prognostic effects of the risk score model were assessed by univariate Cox regression analysis **(A)** and multivariate Cox regression analysis **(B)**. High- and low-risk group risk score rank **(C)**, survival status distribution **(D)**, expression of ferroptosis-related signature **(E)** and principal component analysis (PCA) plots **(F)**.

Both univariate and multivariate analyses were performed to identify prognosis-related factors in the training group (**Figures 5A, B**). Stage, T stage and risk score were considered risk factors in the univariate analysis; however, only the risk score was an independent risk factor in the multivariate analysis. Therefore, the risk score calculated according to the 10-component ferroptosis-related signature was independently associated with the prognosis of patients (HR=3.038, 95%CI=2.023-4.563).

The distribution and status of OS were then analyzed by ranking the risk scores (**Figures 5C, D**). The results showed that patients with higher risk scores were more likely to be deceased. The differential expression profiles of the 10 ferroptosis-related signature are listed in the heatmap of **Figure 5E** between the low-risk group and the high-risk group. The principal component analysis (PCA) also proved that the ferroptosis-related signature prognostic model had the power to distinguish two separate subgroups of HCC patients (**Figure 5F**).

### Verification of the Ferroptosis-Related Signature Model in the Testing Group

**Figure 6:**
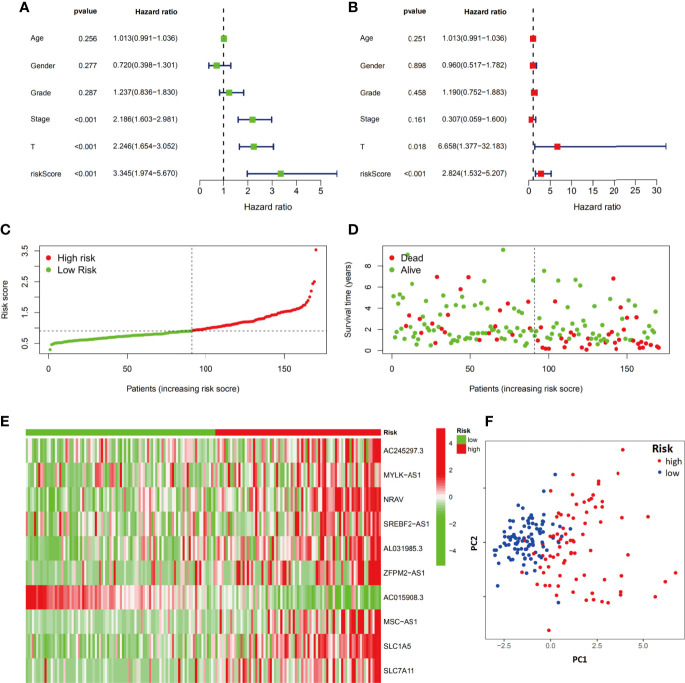
Evaluation of the ferroptosis-related signature in the test group. The independent prognostic value of the risk score model was assessed by univariate Cox regression analysis **(A)** and multivariate Cox regression analysis **(B)**. Risk score rank **(C)**, survival status distribution **(D)**, ferroptosis-related signature component expression **(E)** and PCA plots **(F)** for the high- and low-risk groups.

We next evaluated the prognostic efficiency of the ferroptosis-related signature by analyzing the data in the test cohort. Univariate and multivariate Cox regression analyses were performed to investigate the role of the ferroptosis-related signature in the prognosis of HCC patients (**Figures 6A, B**). Stage, T stage and risk score were risk factors in the univariate regression, and both T stage (HR=6.658, 95%CI=1.377-32.183) and risk score (HR=2.824, 95%CI=1.532-5.207) were further listed as risk factors in the multivariate regression. Similar to the results from the training group, the distribution and status of OS and the expression profiles of the risk-associated ferroptosis-related signature components were also analyzed by ranking the risk scores in the high-risk and low-risk HCC patient groups from the test cohort (**Figures 6C, D**). In addition, the expression trend of 10 ferroptosis-related signature components between the two groups was similar to that of the training group (**Figure 6E**). The PCA plot also showed that the HCC patients were divided into two subgroups by the ferroptosis-related signature model (**Figure 6F**). Overall, the accuracy of the ferroptosis-related signature model was confirmed in the independent validation liver cancer cohorts.

### Evaluation of the Relationship Between Clinicopathological Characteristics and the Ferroptosis-Related Signature

**Figure 7:**
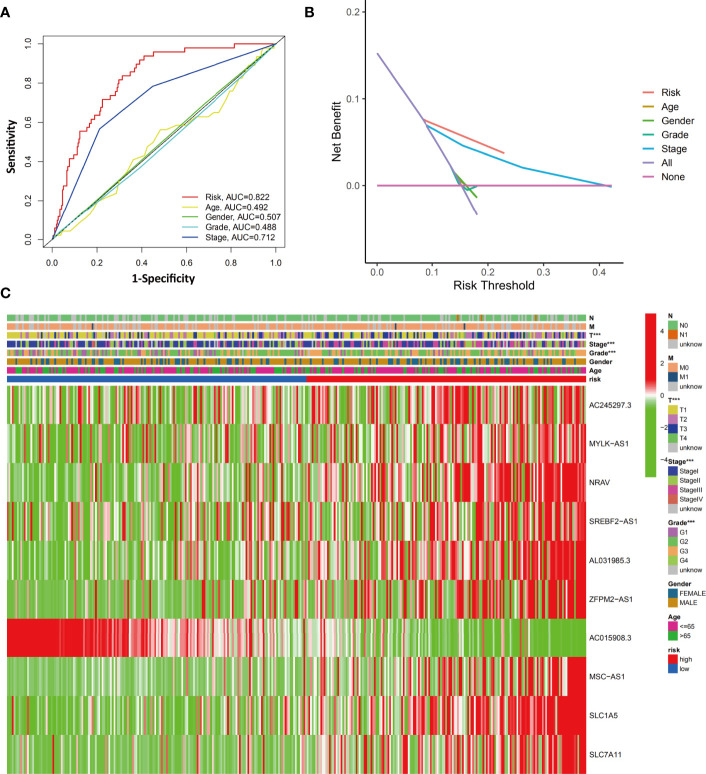
Evaluation of the relationship between clinicopathological characteristics and the ferroptosis-related signature. **(A)** The AUC values of the risk factors and clinicopathological characteristics. **(B)** DCA of the risk factors and clinicopathological characteristics. **(C)** Heatmap of the ferroptosis-related lncRNA prognostic signature and clinicopathological features in HCC patients. ***p < 0.001.

To evaluate the differences between prediction methods, ROC curves were generated for the risk score and clinicopathological characteristics, as shown in **Figure 7A**. The AUC of the ferroptosis-related signature in HCC patients was higher than that of the clinical indexes (AUC=0.822, 1 year). DCA was performed and further showed that the risk score served as a better prognostic indicator than other variables in clinical decision-making (**Figure 7B**).

We next investigated the clinical and pathological features of the low-risk and high-risk groups. The heatmap in **Figure 7C** shows the clinicopathological characteristics in both the high-risk and low-risk groups (grouped according to the ferroptosis-related signature score). The results showed a significant difference between the two groups with respect to HCC grade, stage, and T stage (all p<0.001).

### Differential Immune Cell Infiltration and Function in the Low- and High-Risk Groups

**Figure 8:**
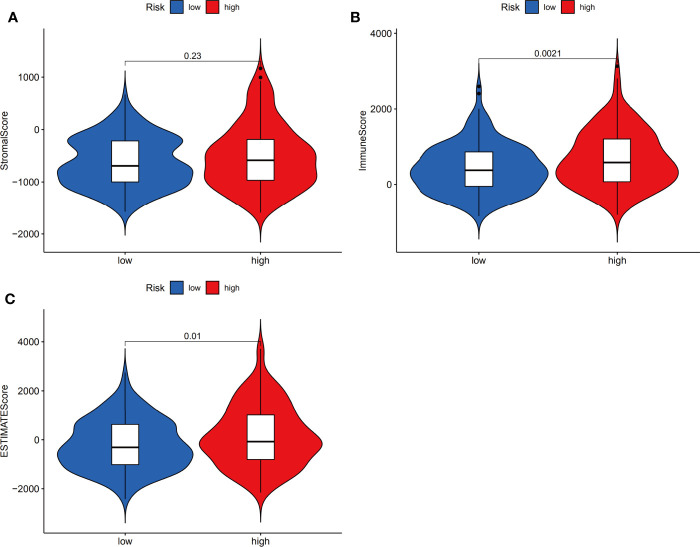
Estimation of stromal and immune cells in malignant tumor tissues using expression data. Violin plots of tumor purity for the low- and high-risk groups according to the stromal score **(A)**, immune score **(B)** and ESTIMATE score **(C)**.

Next, we investigated whether the expression of the ferroptosis-related signature components was associated with the TME. To determine the relationship between the proportion of immune and stromal components and the expression of ferroptosis-related signature components, the stromal score, immune score and ESTIMATE score were assessed in the low-risk and high-risk groups by using the ESTIMATE R package. The results showed no difference in stromal score (**Figure 8A**, *p*=0.23) but showed a significant difference in immune score (**Figure 8B**, *p*=0.0021) and ESTIMATE score (**Figure 8C**, *p*=0.01) between groups.

**Figure 9:**
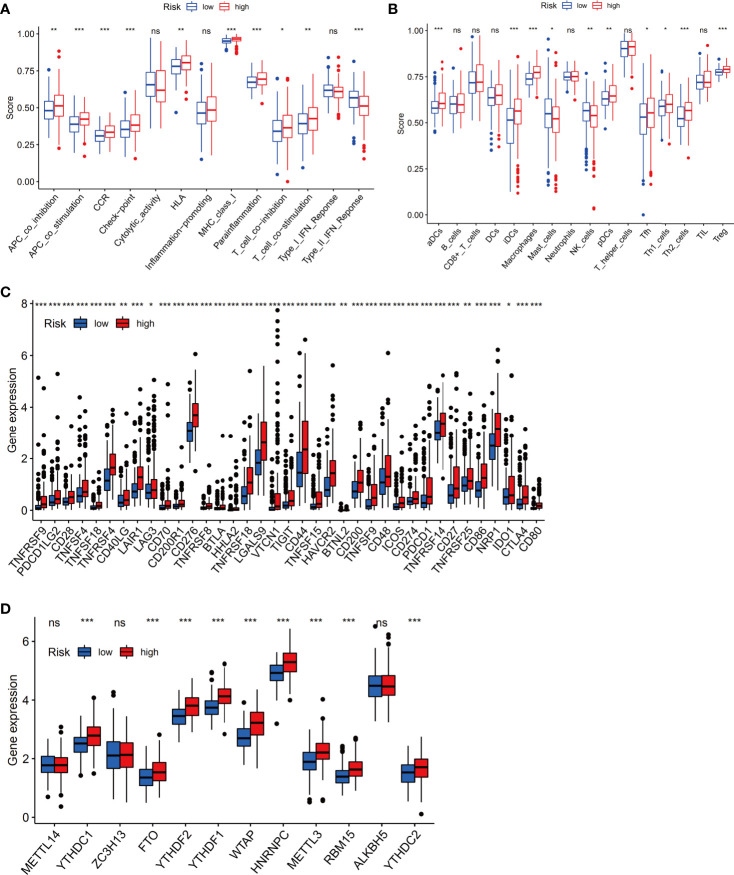
The immune status difference between high-risk and low-risk HCC patients. **(A)** Comparison of immune cell abundance between the high-risk and low-risk groups of HCC patients. **(B)** Comparison of immune-related functions between the high-risk and low-risk groups of HCC patients. **(C)** Comparison of the expression of immune checkpoints between the high- and low-risk groups. **(D)** Comparison of the expression of m6A-associated genes between the high- and low-risk groups. *P < 0.05; **P < 0.01; ***P < 0.001. ns, no significance.

We next evaluated the relationship between immune infiltrates and the ferroptosis-related signature. The results showed activated dendritic cells (aDCs), immature dendritic cells (iDCs), macrophages, Th2 cells and Treg cells were distinct between high- and low-risk groups (all P <0.001). Besides, NK cells, plasmacytoid dendritic cells (pDCs) (both P <0.01), mast cells, T follicular helper (Tfh) cells, Th1 cells (all P <0.05) were also different in two groups (**Figure 9A**). Furtherly, almost all immune-related functions such as APC co-stimulation, APC co inhibition, CCR, and so on, were also different in the two groups (**Figure 9B**). In summary, the results from immune infiltration by ssGSEA showed that the immune status between low- and high-risk groups was totally different, which can be further elucidated to develop tumor immunotherapy in HCC.

Notably, the checkpoint pathway was significantly different between the low-risk and high-risk groups. Considering the clinical potential of checkpoint inhibition for immune therapy, we further explored the difference in the expression of immune checkpoints between the low- and high-risk groups. We observed a statistically significant difference between the two groups in terms of the expression of all checkpoint genes, most of which were more highly expressed in the high-risk group (**Figure 9C**). In addition, we investigated the expression of m6A-related genes between the low-risk group and the high-risk group, and the results showed that the expression of YTHDC1, FTO, YTHDF2, YTHDF1, WTAP, HNRNPC, METTL3, RBM15, and YTHDC2 in the high-risk group was obviously higher than that in the low-risk group (**Figure 9D**).

### Functional Analysis

**Figure 10:**
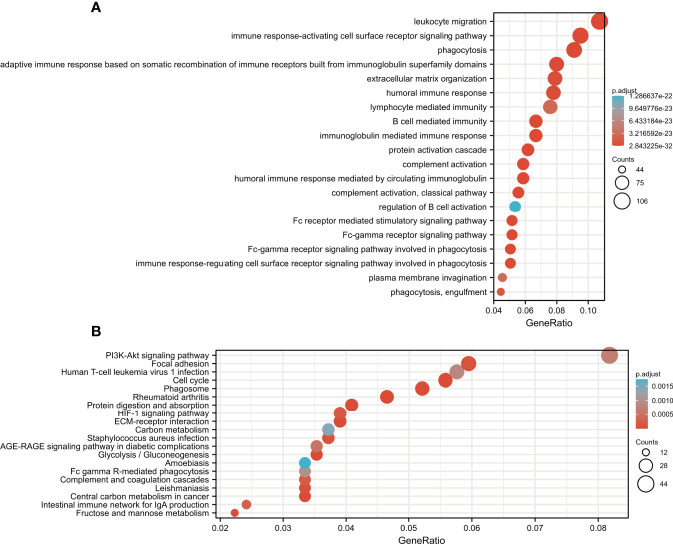
Functional enrichment analysis of the ferroptosis-related signature. **(A)** GO-BP analysis. **(B)** KEGG analysis.

To investigate the biological functions and pathways associated with the risk score, the DEGs between the high-risk and low-risk groups were used to perform GO-BP enrichment and KEGG pathway analyses. Interestingly, the results showed that as many as 856 GO-BP terms and 63 KEGG pathways were identified between low- and high-risk groups (adj *p*<0.05, **Supplementary Table 4** and **Supplementary Table 5**), and the mainly enrichment results were listed in **Figure 10**. As expected, the DEGs were enriched in ferroptosis-associated pathways, such as the PI3K-Akt signaling pathway (31, 32), which is also one of the most frequently altered signaling pathways in human cancers (33–35). On the other hand, the DEGs were also obviously enriched in many immune-related biological processes, such as leukocyte migration, the humoral immune response and B cell-mediated immunity.

### L1000FWD Analysis Identifies Candidate Compounds

**Figure 11:**
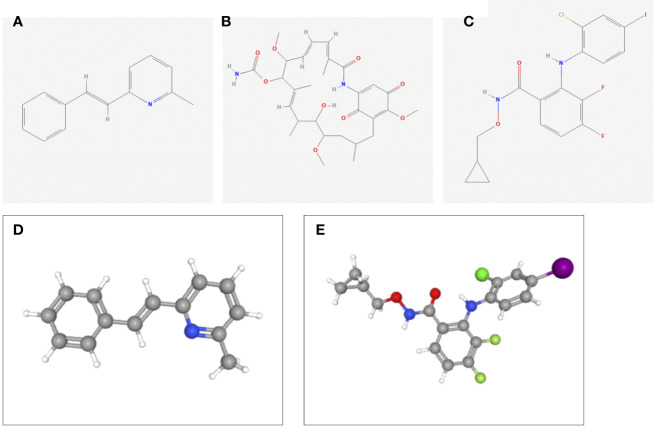
The structure of screened small-molecule compounds. The DEGs were uploaded to L1000FWD website to screen the potential small molecule compounds. The top three compounds were visualized by Pubchem website. **(A)** The 2D structure of SIB-1893; **(B)** The 2D structure of geldanamycin; **(C)** The 2D structure of PD-184352; **(D)** The 3D structure of SIB-1893; **(E)** The 3D structure of PD-184352. The 3D structure of geldanamycin isn’t displayed since too many undefined stereo centers.

To identify the potential drugs for HCC, we uploaded the upregulated and downregulated DEGs to the L1000FWD database. As a result, 10 significant candidate drugs were considered as potential drugs for HCC treatment. The mainly results were showed in **Table 2**. We can discover that these drugs were enriched in the glutamate receptor antagonist, HSP90 inhibitor, MEK inhibitor, c-Met inhibitor and so on. These mechanisms of action and potential small molecule drugs might provide reference for developing potential novel drugs targeting HCC. Among the highly correlated compounds, the structure of SIB-1893, geldanamycin, and PD-184352 were furtherly depicted in **Figure 11**. The 3D structure of geldanamycin isn’t displayed since too many undefined stereocenters.

**Table 2:** The mainly potential drugs identified by L1000FWD database.

Rank	Drug	Similarity Score	p-value	q-value	Z-score	Combined Score	MOA
1	SIB-1893	-0.0763	4.29E-20	2.59E-17	1.67	-32.29	glutamate receptor antagonist
2	geldanamycin	-0.0671	2.45E-16	7.08E-14	1.62	-25.31	HSP inhibitor
3	PD-184352	-0.0624	4.33E-15	9.56E-13	1.75	-25.2	MEK inhibitor
4	PF-04217903	-0.0509	3.27E-09	0.000000191	1.77	-15.06	c-Met inhibitor
5	PD-0325901	-0.0509	2.55E-09	0.000000154	1.68	-14.47	MEK inhibitor
6	FCCP	-0.0486	0.000000042	0.00000183	1.64	-12.06	Unknown
7	PD-198306	-0.0474	3.88E-08	0.00000171	1.68	-12.42	MAP kinase inhibitor, MEK inhibitor
8	BI-2536	-0.0474	0,000000161	0.00000612	1.74	-11.84	PLK inhibitor
9	PAROXETINE	-0.0462	0,000000554	0.0000179	1.63	-10.21	selective serotonin reuptake inhibitor (SSRI)
10	BRD-A66861218	-0.0462	0,000000199	0.00000733	1.76	-11.82	anti-inflammatory agent

## Discussion

In this study, we explored the role of the ferroptosis-related signature, which includes mRNAs and lncRNAs, in HCC. A prognostic model including 10 ferroptosis-related mRNAs/lncRNAs was first constructed and tested in the TCGA-LIHC dataset. Furthermore, immune analysis, including analysis with various bioinformatics tools, indicated obvious differences in the TME and immune cell infiltration between the low-risk and high-risk groups, especially in terms of checkpoint genes and m6A-associated genes. Functional analysis revealed that many tumor-related pathways were enriched. These findings strongly implied the great potential roles of ferroptosis in HCC.

The rapid progression of cancer is accompanied by the transformation and acceleration of a variety of metabolic pathways, which often means that a large number of metabolic byproducts, such as oxygen free radicals, accumulate in tumor cells to activate the oxidative stress pathway. Although tumor cells should have been vulnerable to ferroptosis, it has been found that cancer cells can acquire their resistance to ferroptosis through alteration of gene expression (36, 37). However, reactivation of the ferroptosis-related pathway in tumor cells may provide new therapeutic targets for tumor therapy. Although current therapeutic strategies, including surgical intervention, tumor-targeted drugs, immunotherapeutic agents or antiviral treatment, have improved OS in HCC patients in an inspiring way, their applications are still limited by tumor heterogeneity (38) and the development of drug resistance (39, 40).

The prognostic model in our study integrated 10 ferroptosis-related components, including 2 genes (SLC1A5 and SLC7A11) and 8 lncRNAs (AC245297.3, MYLK-AS1, NRAV, SREBF2-AS1, AL031985.3, ZFPM2-AS1, AC015908.3, MSC-AS1). Solute carrier family 1 member 5 (SLC1A5) is reported to be a driver gene of ferroptosis that mediates the uptake of glutamine, a conditionally essential amino acid in rapidly proliferating tumor cells (41, 42). In erastin- and RSL3-induced ferroptosis, glutamine importation and metabolism induce lipid ROS generation and promote cell death (43). Suppression of SLC1A5 by miR-137 or the small molecular inhibitor GPNA strongly inhibits glutaminolysis to cause ferroptotic cell death (44). As SLC1A5-mediated glutamine transport plays a key role in tumor cell metabolism, proliferation, and ferroptosis, blocking SLC1A5 has been shown to successfully prevent tumor cell proliferation in melanoma (45), non-small-cell lung cancer (46, 47), prostate cancer (48) and acute myeloid leukemia (49). Solute carrier family 7 member 11 (SLC7A11) is a pivotal protein component of system Xc- that is responsible for maintaining redox homeostasis by importing cystine, where it is then reduced to cysteine and used to synthesize the major antioxidant GSH (50, 51). Numerous experiments have demonstrated its high expression in various cancers and multiple effects on cancer growth, invasion, metastasis and unfavorable prognosis (52–60). It has been demonstrated that SLC7A11 confers resistance to ferroptosis in cancer cells by importing cystine for the synthesis of GSH and indirectly relieving lipid ROS stress by activating the essential enzyme GPX4 to reduce lipid hydroperoxides (50, 61). Moreover, SLC7A11 has been demonstrated to be involved in the resistance to anticancer treatments, which was supported by experiments in which therapeutic resistance was reversed by directly targeting SLC7A11 (59, 60, 62–66). Therefore, targeting SLC7A11 exhibits good potential for the treatment of cancer, and several drugs targeting SLC7A11 are being prepared for clinical testing (54–58).

As the factor with the highest positive correlation coefficient in the prognostic model, lncRNA MSC-AS1 has been reported to be involved in HCC (67), lung adenocarcinoma (68), laryngeal cancer (69) and kidney renal clear cell carcinoma (70). It acts sponging miR-33b-5p to upregulate GPAM (68). Another study indicated that it activates the Wnt/β-catenin pathway to regulate tumor proliferation and migration *via* miR-3924/WNT5A (70). MYLK-AS1 was reported to be associated with tumor progression and angiogenesis in HCC. The mechanism involves targeting the miR-424-5p/E2F7 axis and activating the VEGFR-2 signaling pathway (71) or stimulating the EGFR/HER2-ERK1/2 signaling pathway (72). LncRNA NRAV has been reported to be involved in the antiviral immune response (73, 74). In addition, several independent bioinformatics analyses have demonstrated that it is a valuable clinical prognostic biomarker in HCC (75, 76) and lower-grade glioma (77). ZFPM2-AS1 is reported to be involved in numerous tumors, including lung adenocarcinoma (78), renal cell cancer (79), gastric carcinoma (80) and HCC (81). Mechanistically, it is reported to attenuate the p53 pathway by stabilizing MIF (80) or regulating miR-139/GDF10 (81).

Few studies have investigated lncRNA MSC-AS1, AL031985.3, and AC245297.3. However, they are indicated by different bioinformatics analyses to have prognostic value [MSC-AS1 (82, 83), AL031985.3 (84–86) in HCC, and AC245297.3 (87, 88) in breast cancer]. Therefore, these factors may be involved in multiple mechanisms in HCC and breast cancer, and their specific regulation mechanisms still further study. In particular, MSC-AS1 was the only protective lncRNA in the model and had a high correlation coefficient. There are no studies on lncRNA SREBF2-AS1 at present, so it needs further study. Notably, although several identified ferroptosis-related lncRNAs have not been well studied, in our study, the lncRNAs were closely co-expressed with many ferroptosis-related genes (**Figure 3C**).

The results from both immune infiltration analysis and enrichment analysis indicate that a higher level of APCs and humoral immunity including B cells and the complement system in the high-risk group. Besides, results from both difference in APC co-stimulation and co-inhibition appears contradictory in the immune microenvironment. However, we found the results are somewhat similar to the researches about ferroptosis-related signature in other cancers (6, 89–91). Though the mechanisms illustrating tumor susceptibility to ferroptosis have been an intense area of research in past decades, the complex relationship between tumor immunity and ferroptosis remains elusive. Tumor-infiltrating lymphocytic B cells (TIL-B) have been reported to be a main component of TILs in ovarian and breast cancers, which may be correlated with improved survival (92, 93). However, tumor development is enhanced when B cells are present have been reported in several mouse models (94, 95). For pancreatic carcinoma, several researches have demonstrated that TIL-Bs in supporting both early and more advanced stages of pancreatic tumorigenesis by multiple mechanisms, including suppression of other immune cells (e.g., CD8+ T cells and macrophages) in the tumor microenvironment and promoting pancreatic cancer cell proliferation (96).

Based on the DEGs between high- and low-risk groups, several small molecule drugs targeting ferroptosis-related signature components were identified *via* the L1000FWD database which was mainly enriched in the glutamate receptor antagonist, HSP90 inhibitor, MEK inhibitor and so on. As described above, glutamate metabolism was involved in the SLC1A5-induced ferroptosis, which may be inhibited by glutamate receptor antagonist, such as SIB-1893. HSP90-associated chaperone-mediated autophagy has been demonstrated to obviously promote ferroptosis (97, 98), which could be reduced by geldanamycin. The activation of the Raf-MEK-ERK pathway plays an important role in the proliferation, differentiation, invasion and metastasis of cancer cells (99). PD-184352 was the first MEK inhibitor to enter the clinical trial. It was terminated in phase II clinical trial because of its poor solubility, low oral bioavailability and large individual differences (100). Though the application of MEK inhibitor is limited by clinical efficacy and drug resistance, the development of novel MEK inhibitor is still one of the promising directions of anti-cancer treatment (101–103).

The model exhibited several advantages. 1) The prognostic model showed excellent performance in the ROC curve analysis, with AUCs as high as 0.830 and 0.806 in the training and test groups, respectively. As depicted in the DCA, the ferroptosis-related signature had better predictive value than all pathological characteristics. The risk score was identified as an independent risk factor, with HR=3.038 in the training group and 2.824 in the test group. Therefore, the model showed excellent clinical prognostic value. 2) According to the risk score from the model, the HCC patients were divided into two distinct groups. Analysis of the TME and immune infiltration showed that there were numerous variables with statistically significant differences between the two groups, especially the expression of checkpoint genes. These differences were consistent with the differences observed in the GO and KEGG analysis. Therefore, immunotherapies targeting the 10 components of the ferroptosis-related signature are promising methods for anti-tumor treatment. 3) Considering that there are frequent updates in the field of ferroptosis research, a large number of studies and ferroptosis-related databases were assessed (12, 13, 28–30). As many as 267 candidate ferroptosis-related genes were included in the analysis. To our knowledge, this is the largest ferroptosis-related gene list used for bioinformatics analysis at present.

There are also some limitations to this study. Due to the limited knowledge of ferroptosis, most of the signature components in our research are involved not only in ferroptosis-related pathways but also in other pathways, such as immunity and autophagy. This may be more common for lncRNAs, which have nonspecific functions in biological processes. Therefore, it is difficult to evaluate the exact role of ferroptosis alone in HCC with our risk score. These results should be further validated in external HCC cohorts from multicenter research. In another attempt to investigate the role of ferroptosis-related in other cancers, we performed the survival analysis of SLC7A11, MSC-AS1 and MYLK-AS1 in gastrointestinal cancer including colon cancer, esophageal cancer, liver cancer, pancreatic cancer and stomach cancer (**Supplementary Figure**). Most of results showed that they are only play a specific role in liver cancer.

In conclusion, our research established a 10-component ferroptosis-related signature including mRNAs and lncRNAs for predicting the prognosis of HCC patients. The ferroptosis-related signature showed excellent performance in predicting clinical prognosis. The signature can be used to calculate the risk score, which accurately reflects the tumor environment and immune filtration of patients, thereby providing a reference for clinical treatment. Therefore, the ferroptosis-related signature is expected to be a new biomarker for both diagnosis and treatment decision making. Further investigation of the role and mechanism of the 10-component ferroptosis-related signature in the progression of HCC is still needed.

## Data Availability Statement

The original contributions presented in the study are included in the article/**Supplementary Material**. Further inquiries can be directed to the corresponding authors.

## Author Contributions

Z-AC collected the papers and analyzed data, analyzed the conclusions, and drafted the manuscript. HT reviewed the data and conclusions. D-MY and YZ contributed to writing. C-JY and Z-JF presented the idea of this manuscript, supported the funding, analyzed the conclusions, drafted and revised the manuscript. All authors contributed to the article and approved the submitted version.

## Funding

This study was funded by the Natural Science Foundation of Hebei Province (Grant No. H2020206337).

## Conflict of Interest

The authors declare that the research was conducted in the absence of any commercial or financial relationships that could be construed as a potential conflict of interest.

## Publisher’s Note

All claims expressed in this article are solely those of the authors and do not necessarily represent those of their affiliated organizations, or those of the publisher, the editors and the reviewers. Any product that may be evaluated in this article, or claim that may be made by its manufacturer, is not guaranteed or endorsed by the publisher.
