# Supplementary material for: Identification of a Ferroptosis-Related Signature Model Including mRNAs and lncRNAs for Predicting Prognosis and Immune Activity in Hepatocellular Carcinoma
Source: Front Oncol. 2021 Sep 9;11:738477. doi: 10.3389/fonc.2021.738477 (PMC8458836; doi:10.3389/fonc.2021.738477)
Supplement: Supplementary file 1 [file DataSheet_1.docx]

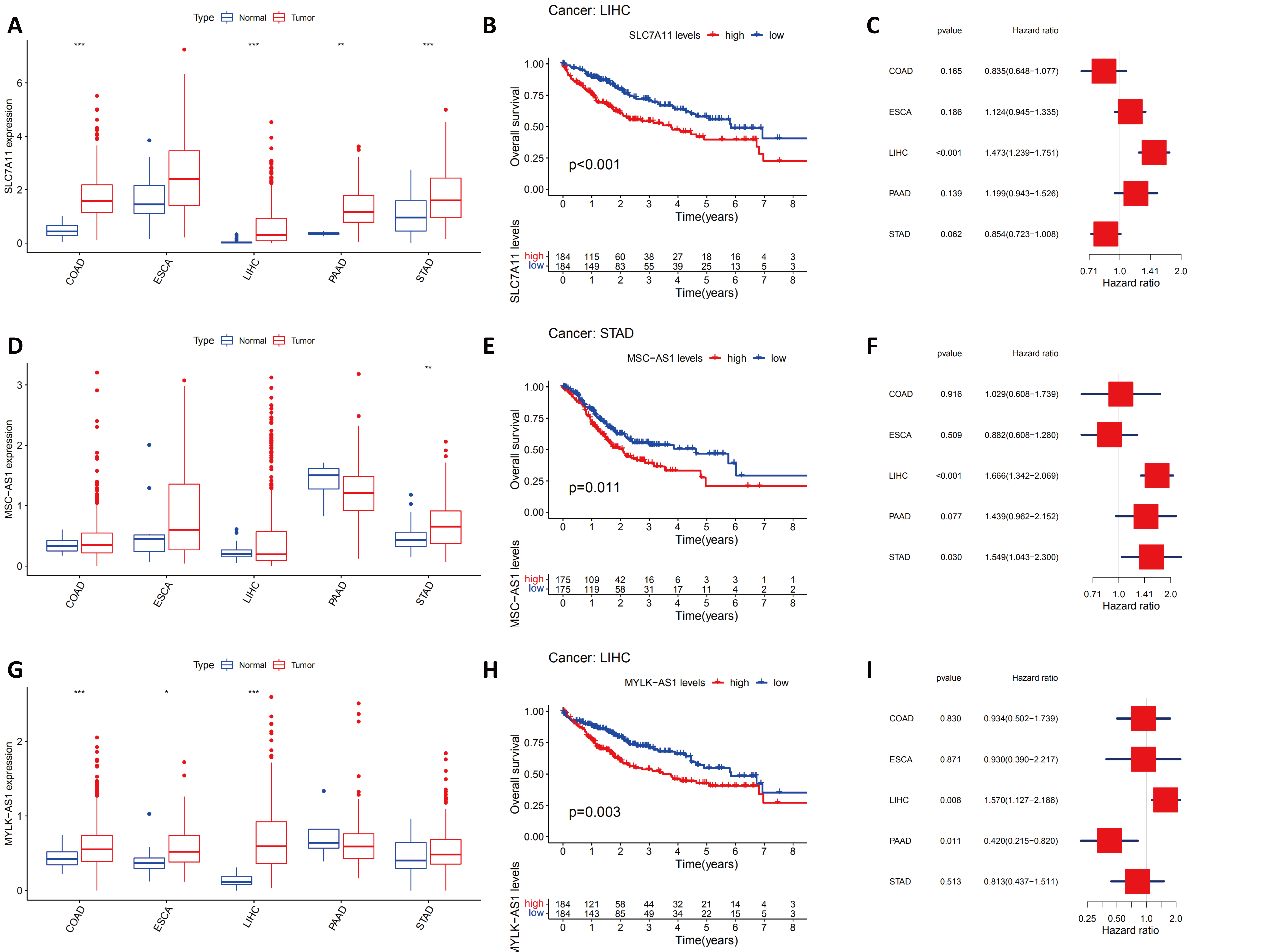


**Supplementary Figure** The role of SLC7A11, MSC-AS1 and MYLK-AS1 in gastrointestinal cancer (COAD, ESCA, LIHC, PAAD, and STAD). (A) The expression of the SLC7A11 between normal group and cancer group in COAD, ESCA, LIHC, PAAD, and STAD, respectively. (B) The K-M survival analysis for SLC7A11 were performed in five gastrointestinal cancer cohort, and only in the LIHC cohort showed a statistical difference. (C) The univariate cox regression analysis of SLC7A11 were performed in five gastrointestinal cancer cohort. (D) The expression of the MSC-AS1 between normal group and cancer group in COAD, ESCA, LIHC, PAAD, and STAD, respectively. (E) The K-M survival analysis for MSC-AS1 were performed in five gastrointestinal cancer cohort, and only in the STAD cohort showed a statistical difference. (F) The univariate cox regression analysis of MSC-AS1 were performed in five gastrointestinal cancer cohort. (G) The expression of the MYLK-AS1 between normal group and cancer group in COAD, ESCA, LIHC, PAAD, and STAD, respectively. (H) The K-M survival analysis for MYLK-AS1 were performed in five gastrointestinal cancer cohort, and only in the LIHC cohort showed a statistical difference. (I) The univariate cox regression analysis of MYLK-AS1 were performed in five gastrointestinal cancer cohort. COAD: colon cancer; ESCA: [Esophageal Cancer](https://xenabrowser.net/datapages/?cohort=GDC TCGA Esophageal Cancer (ESCA)&removeHub=https://xena.treehouse.gi.ucsc.edu:443); LIHC: [Liver Cancer](https://xenabrowser.net/datapages/?cohort=GDC TCGA Liver Cancer (LIHC)&removeHub=https://xena.treehouse.gi.ucsc.edu:443); PAAD: [Pancreatic Cancer; STAD:](https://xenabrowser.net/datapages/?cohort=GDC TCGA Pancreatic Cancer (PAAD)&removeHub=https://xena.treehouse.gi.ucsc.edu:443) [Stomach Cancer](https://xenabrowser.net/datapages/?cohort=GDC TCGA Stomach Cancer (STAD)&removeHub=https://xena.treehouse.gi.ucsc.edu:443)[. ***p < 0.001, **p < 0.01, *p < 0.05.](https://xenabrowser.net/datapages/?cohort=GDC TCGA Pancreatic Cancer (PAAD)&removeHub=https://xena.treehouse.gi.ucsc.edu:443)
